# Radiomics features of the primary tumor fail to improve prediction of overall survival in large cohorts of CT- and PET-imaged head and neck cancer patients

**DOI:** 10.1371/journal.pone.0222509

**Published:** 2019-09-19

**Authors:** Rachel B. Ger, Shouhao Zhou, Baher Elgohari, Hesham Elhalawani, Dennis M. Mackin, Joseph G. Meier, Callistus M. Nguyen, Brian M. Anderson, Casey Gay, Jing Ning, Clifton D. Fuller, Heng Li, Rebecca M. Howell, Rick R. Layman, Osama Mawlawi, R. Jason Stafford, Hugo Aerts, Laurence E. Court

**Affiliations:** 1 Department of Radiation Physics, The University of Texas MD Anderson Cancer Center, Houston, Texas, United States of America; 2 MD Anderson Cancer Center UTHealth Science Center at Houston Graduate School of Biomedical Sciences, Houston, Texas, United States of America; 3 Department of Biostatistics, The University of Texas MD Anderson Cancer Center, Houston, Texas, United States of America; 4 Division of Radiation Oncology, The University of Texas MD Anderson Cancer Center, Houston, Texas, United States of America; 5 Department of Imaging Physics, The University of Texas MD Anderson Cancer Center, Houston, Texas, United States of America; 6 Department of Radiation Oncology, Dana Farber Cancer Institute, Brigham and Women’s Hospital, Harvard Medical School, Boston, Massachusetts, United States of America; Chang Gung Memorial Hospital at Linkou, TAIWAN

## Abstract

Radiomics studies require many patients in order to power them, thus patients are often combined from different institutions and using different imaging protocols. Various studies have shown that imaging protocols affect radiomics feature values. We examined whether using data from cohorts with controlled imaging protocols improved patient outcome models. We retrospectively reviewed 726 CT and 686 PET images from head and neck cancer patients, who were divided into training or independent testing cohorts. For each patient, radiomics features with different preprocessing were calculated and two clinical variables—HPV status and tumor volume—were also included. A Cox proportional hazards model was built on the training data by using bootstrapped Lasso regression to predict overall survival. The effect of controlled imaging protocols on model performance was evaluated by subsetting the original training and independent testing cohorts to include only patients whose images were obtained using the same imaging protocol and vendor. Tumor volume, HPV status, and two radiomics covariates were selected for the CT model, resulting in an AUC of 0.72. However, volume alone produced a higher AUC, whereas adding radiomics features reduced the AUC. HPV status and one radiomics feature were selected as covariates for the PET model, resulting in an AUC of 0.59, but neither covariate was significantly associated with survival. Limiting the training and independent testing to patients with the same imaging protocol reduced the AUC for CT patients to 0.55, and no covariates were selected for PET patients. Radiomics features were not consistently associated with survival in CT or PET images of head and neck patients, even within patients with the same imaging protocol.

## Introduction

The process of radiomics involves evaluating images on a voxel level to extract quantitative image features (i.e., texture). These radiomics features, combined with conventional prognostic factors (e.g., age), have improved patient outcome models, increasing the interest in radiomics studies [1–5].

Studies using computed tomography (CT) images from patients with head and neck cancer have found that radiomics features were significantly associated with local control, tumor failure, overall survival, and human papillomavirus (HPV) status [6–12]. Similar findings from positron-emission tomography (PET) images of head and neck cancer patients have shown that radiomics features were significantly associated with local control, tumor failure, overall survival, and freedom from distant metastases [10, 12–14].

However, studies have shown that imaging protocol differences, such as pixel size can increase uncertainties in patient datasets [15–19]. A recent phantom study showed that inter-scanner variability can be reduced by more than 50% when a controlled imaging protocol is used for CT imaging [19]. For PET images, acquisition and reconstruction parameters have been shown to affect radiomics features; particularly, the number of iterations, matrix size, and smoothing filter have demonstrated variability [20–24].

Based on these uncertainty studies, our hypothesis is that outcome models built with data from patients on controlled imaging protocols should perform better than models built with data from a varied patient cohort since the noise from imaging variability is removed in the former model. We aimed to test this hypothesis in large cohorts of CT and PET head and neck cancer patients.

## Materials and methods

### CT patients

Patients who were treated with definitive radiotherapy for head and neck squamous cell carcinoma (HNSCC) at least five years ago, had pre-treatment CT images available, did not have a tumor stage of Tx (primary tumor could not be assessed), T0 (no evidence of primary tumor), or Tis (carcinoma *in situ*), and did not have a nodal stage of Nx (regional lymph nodes could not be assessed) were considered eligible. We retrospectively reviewed contrast-enhanced pretreatment CT images and medical records of 652 patients with oropharyngeal HNSCC that were treated between March 2004 and November 2013 with a waiver of informed consent and study approval from the Institutional Review Board at The University of Texas MD Anderson Cancer Center. All patients were scanned on GE scanners (GE Healthcare, Chicago, IL). The primary gross tumor volume (GTV) was contoured by two radiation oncologists specific for this study. In addition, 156 HNSCC patients from Aerts et al.’s data set from MAASTRO were included [6]. Fifty patients were excluded from this data set due to no contoured GTV, other missing data elements, or issues with importing data into our radiomics software.

Patients whose GTV was more than 50% affected by streak artifacts were excluded from our study. Our previous work has shown that this cutoff was useful for including only those patients whose features from GTV not affected by streak artifacts represented features from the whole GTV [25]. Removing all patients with any streak artifact within their GTV would have removed 215 patients. Therefore, this method allows many more patients to be included in the study, as this resulted in the removal of only 32 patients from the study, while not impacting feature values. The remaining 726 patients were divided into training and independent testing cohorts by medical record number (MRN): those with an odd MRN were placed into the training cohort (377 patients), and those with an even MRN were placed into the independent testing cohort (349 patients). The patient demographics for each cohort are summarized in Table 1. Independent testing is used here to identify that these patients were not used during the training loops and were only used during the final evaluation of the built model.

**Table 1:** Patient demographics.

	CT Patients		PET Patients	
	Training Cohort	Testing Cohort	Training Cohort	Testing Cohort
Number of patients	377	349	345	341
Number of events	97	75	76	51
Age (years)*	59 (21–87)	57 (30–80)	60 (34–87)	58 (35–90)
HPV status				
Positive	224	189	207	206
Negative/unknown	153	160	138	135
Tumor stage				
T1	71	78	52	63
T2	143	142	131	142
T3	88	72	111	75
T4	75	57	51	61
Nodal stage				
N0	47	40	47	38
N1	34	34	39	40
N2	286	260	248	245
N3	10	15	11	18
AJCC stage				
I-II	20	20	18	21
III	48	45	57	52
IV	309	284	270	268
Primary Gross Tumor Volume (cm^3^)*	9 (0.3–326)	8 (0.3–150)	9 (0.8–81)	9 (0.4–123)

* median; range in parentheses

Our previous work has shown that inter-scanner variability can be significantly reduced when using a controlled protocol [19]. To investigate this impact on the prognostic ability of patient outcome models, we included in these cohorts only patients who had been scanned on a GE scanner with a standard kernel, 1.25-mm image thickness, and 25-cm field of view because the largest subset cohort could be created from the original cohort using these settings. Most of the acquisition parameters have been shown to not impact features, while these reconstruction parameters (kernel, image thickness, and field of view) have been shown to affect features [16–18, 26, 27]. Thus we focused reconstruction parameters for selecting the subset of patients. These patients were only from MD Anderson as the MAASTRO data was not on a GE scanner.

### PET patients

Patients who were treated with definitive radiotherapy for HNSCC at least four years ago, had pre-treatment PET images available, did not have a tumor stage of Tx (primary tumor could not be assessed), T0 (no evidence of primary tumor), or Tis (carcinoma *in situ*), and did not have a nodal stage of Nx (regional lymph nodes could not be assessed) were considered eligible. We retrospectively reviewed the images and medical records of 445 patients with oropharyngeal HNSCC that were treated between March 2004 and November 2013 with a waiver of informed consent and study approval from the Institutional Review Board at The University of Texas MD Anderson Cancer Center. In addition, we used images, patient survival data, and demographics from the Head-Neck-PET-CT TCIA collection [28, 29]. This collection contained 298 patients, 241 of whom were included; those excluded had lesions with no F18-FDG PET radiotracer uptake or there were issues with importing data into our radiomics software. Each patient’s primary GTV was contoured using MIM PET Edge (MIM Software Inc, Cleveland, OH).

The 686 patients were divided into training and independent testing cohorts by MRN: those with an odd MRN were placed into the training cohort (345 patients), and those with an even MRN were placed into the independent testing cohort (341 patients). The patient demographics for each cohort are summarized in Table 1.

To investigate the effect of reducing inter-scanner variability on the predictive performance of patient outcome models, we included in these cohorts only patients who had been scanned on a GE scanner with two iterations and 20 or 21 subsets; these reconstruction settings were chosen to enable the largest subset cohort to be created from the original cohort. Additionally, in our unpublished work we have found that iterations and subsets can cause the largest discrepancies in radiomics features from the reconstruction parameters that can be changed. However, inter-vendor variances can be large, thus restricting this subset to only patients imaged on a GE scanner is the main driving force in reducing the uncertainty for this study.

### Feature extraction

The radiomics features were calculated using IBEX, an open-source radiomics tool [30, 31]. Tables of the extracted features are provided in the Supplemental Material. The settings for each feature were the same as those listed in Fave et al.’s Supplemental Material [1]. All of the features were calculated by using four different preprocessing techniques for the CT images: (1) thresholding (lower limit -100 HU, no upper limit), (2) thresholding and a Butterworth smoothing filter (order of 2, cut-off of 125), (3) thresholding and 8-bit depth resampling, and (4) thresholding, 8-bit depth resampling, and Butterworth smoothing. Different features have been shown to be most prognostic with different preprocessing techniques, which is why this assortment of preprocessing techniques was chosen [32]. For the PET images, all of these features were preprocessed using two methods: (1) a fixed bin width of 0.5 SUV, as suggested by Leijenaar et al. [33], and (2) rescaling to 64 levels, as suggested by Hatt et al. [34]. The volume of each GTV was also extracted.

### Model building

The modeling process used here is based on that used for several of our previous, successful radiomics studies [1–3]. The overall survival was defined as the time interval from the end of definitive radiotherapy to death, and was censored at the last follow-up for patients who were alive. The end point of overall survival was selected for this study because the number of events are higher than events using locoregional control or freedom from distant metastases as an end point. The model was built by using scaled training data and then fitting the final model with the non-scaled training data and computing receiver operator curve statistics by using the trained model on the independent testing data. The radiomics features and volume of the training data were scaled by subtracting the mean and dividing by the standard deviation for each since Lasso penalizes larger values more (R version 3.5.1). Tumor volume and HPV status were the only clinical variables used in order to focus on the effect of the radiomics features. Tumor volume was a surrogate for T stage in this study as T stage is primarily determined by the size of the tumor [35], but volume is a continuous variable allowing for more finite discrimination.

To begin building the model, we first used univariate Cox proportional hazards models to select the one preprocessing technique for each feature that had the most significant association with overall survival. Clinical variables were used in forward selection, keeping only those that reduced the Akaike information criteria (AIC) by more than 2. Next, the selected clinical variable(s) were held constant in a univariate Cox proportional hazards model with the prescreened radiomics features to further reduce the dimensionality of the data (R survival package version 2.42–6). The features that had a p-value less than 0.01 were kept. One thousand bootstrap iterations of Lasso regression, using the selected radiomics features and clinical variables, were conducted (R glmnet package version 2.0–16). For these 1000 iterations, the Lasso was fit by using the minimum lambda determined from a 10-fold cross-validation with a maximum of 1000 iterations. The covariates selected in more than 50% of the 1000 bootstrap iterations were kept. Due to the minimum lambda under penalizing the regression, a final forward selection was performed. Those covariates that reduced the AIC by more than 2 were selected. A final Cox model was fit by using these covariates and the non-scaled training data.

The area under the curve (AUC) of the final Cox model when predicting overall survival in the independent testing data was calculated at 3 years (R survivalROC package version 1.0.3). Patients were assigned to the “High Risk” group if their prediction score was higher than the median; otherwise they were assigned to the “Low Risk” group. The survival probability curve of each group was estimated by the Kaplan-Meier method. The separation between these groups was evaluated by the log-rank test and determined to be significant if the p-value was less than 0.05 (R survival package). Models were built separately for the whole patient cohort and the subset of patients with the same imaging protocol.

We also examined the HPV positive and negative/unknown patients separately because HPV status is a strong known predictor of overall survival. Additionally, most of the patients in our original patient cohort had oropharyngeal cancer, therefore, we analyzed the data using only these patients as well. For these subgroups, the whole modeling process was repeated, including modeling with only those patients with the same imaging protocol to allow for comparisons.

## Results

### CT patients

**Fig 1:**
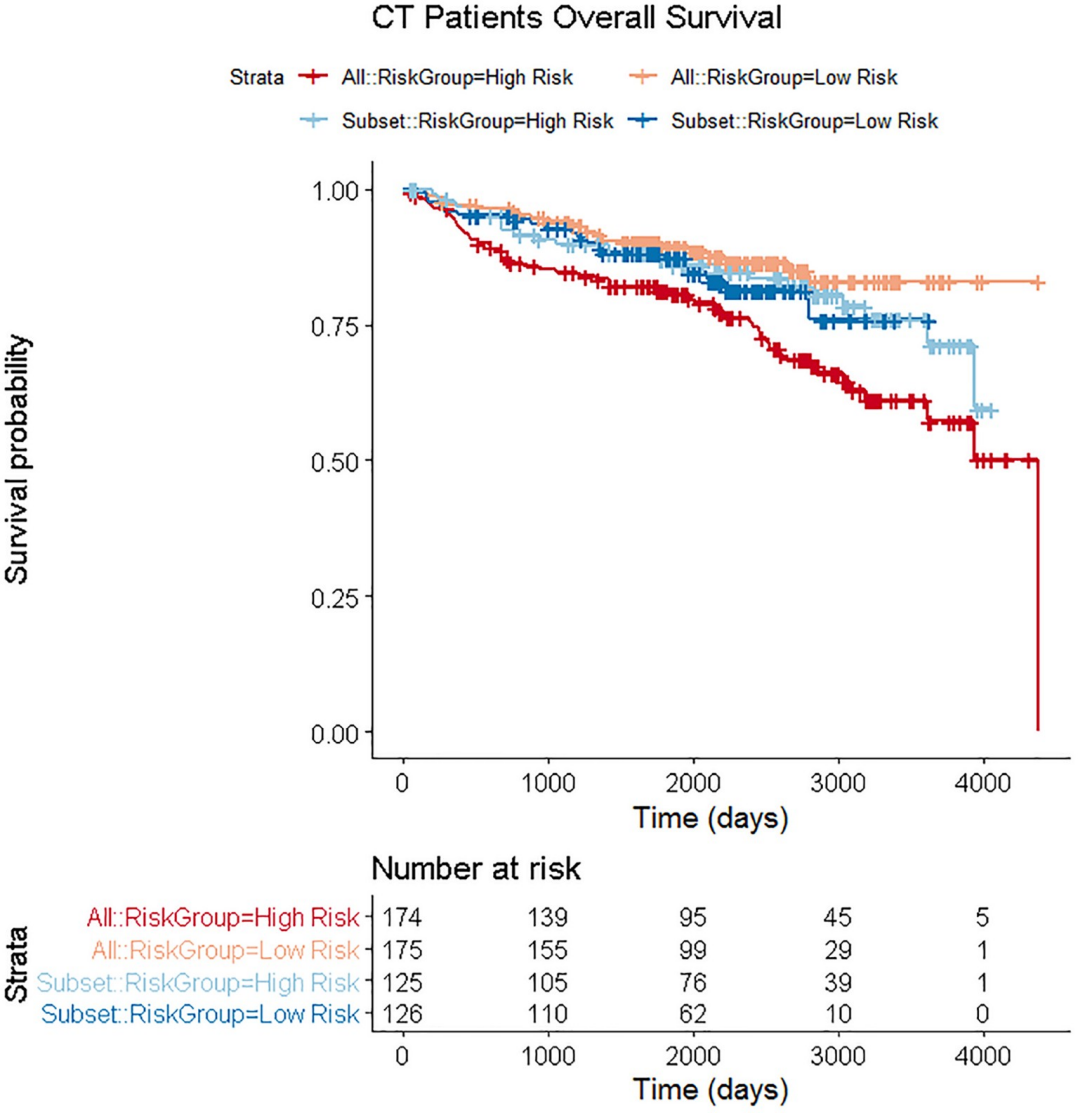
Patient survival curves using CT patient data for the cohort using all patients and the subset of patients that had the same imaging protocol. For the cohort using all patients, the independent testing data were from 349 patients who were assigned to High Risk or Low Risk groups according to prediction scores from the Cox model fit using the training data and the four covariates: volume, HPV status, gray level nonuniformity calculated using thresholding and bit depth resampling, and inverse difference norm calculated using thresholding. The separation between the curves was statistically significant (p = 5x10^-4^). These patient curves are called “All” and are in red and orange. For the subset of patients with the same imaging protocol, the independent testing data were from 251 patients who were assigned to High Risk or Low Risk groups according to prediction scores from the Cox model fit using the training data and the two covariates: HPV status and cluster tendency calculated using thresholding, smoothing, and bit depth resampling. The separation between the curves was not statistically significant. These patient curves are called “Subset” and are in blue and light blue.

When using the whole patient cohort, volume and HPV status were selected from the forward selection of the clinical variables. Twelve radiomics features had a p-value < 0.01 when tumor volume and HPV status were held within the Cox proportional hazards model. Five covariates were selected from the bootstrap Lasso. The final selected model contained the following four covariates: tumor volume, HPV status, gray level nonuniformity calculated using thresholding and bit depth resampling, and inverse difference norm calculated using thresholding. The AUC of this model on the independent testing data was 0.72. The High Risk and Low Risk groups were statistically separated (p = 5x10^-4^). Survival plots are shown in Fig 1. However, when a Cox model with these covariates was fit on the independent testing data, volume, gray level nonuniformity, and inverse difference norm were just under the significance threshold (p = 0.027, p = 0.024, and p = 0.017, respectively), and HPV status was not significant (p = 0.18), although all four covariates were significant in univariate models on the independent testing data. Volume alone or volume and HPV status fit in a Cox model on the training data and evaluated on the independent testing data provided an AUC of 0.73. Adding any radiomics features to this reduced the AUC. This is interesting as other covariates were significant in the multi-variable Cox model. However, the covariates were not strongly significant in the multi-variable Cox model, while the p-value of volume in the Cox model lowered to 6.08 x 10^−9^ when it was alone which is likely the reason for the increase in AUC with volume alone.

The CT imaging protocol is also known to affect radiomics features measured from CT images [15–19]. Therefore, to reduce the noise in the data sets, only those patients scanned on a GE scanner with the same imaging protocol were included. This reduced the training data to 260 patients and the independent testing data to 251 patients. The final model using this data included two covariates and had an AUC of 0.55 on the independent testing data. However, neither covariate was significant (p = 0.90, p = 0.79) in the independent testing data, so this attempt to control for imaging parameters was not effective. The High Risk and Low Risk groups were not statistically separated, and the survival curves for these risk groups are shown in Fig 1 alongside the survival curves using all of the patients. Table 2 summarizes the model information for the whole patient data set and the subset of patients with the same imaging protocol.

**Table 2:** Model information for CT and PET patients.

Patient Information				Model Information		Evaluation Information	
Image Type	Subset of Patients	Patients in training	Patients in testing	Covariates in final model	Hazard ratio of covariates on training data (95% CI)	p-value of covariates when fit on testing data	AUC on testing data
CT	All patients	377	349	Volume	1.01 (1.00–1.02)	p = 0.027	0.72
				HPV status	1.93 (1.27–2.95)	p = 0.18	
				Gray level nonuniformity (GLCM) calculated using thresholding and bit depth resampling	9.74 x 10^−8^ (9.22 x 10^−12^–1.03 x 10^−3^)	p = 0.024	
				Inverse difference norm (HLCM) calculated using thresholding	3.34 x 10^6^ (13.5–8.28 x 10^11^)	p = 0.017	
CT	Same imaging protocol	260	251	HPV status	2.27 (1.32–3.89)	p = 0.79	0.55
				Cluster tendency (GLCM) calculated using thresholding, smoothing, and bit depth resampling	1.07 (1.04–1.11)	p = 0.90	
PET	All patients	345	341	HPV status	1.8 (1.14–2.9)	p = 0.69	0.59
				Coarseness calculated using 64 gray levels	2614 (11.6–5.9 x 10^5^)	p = 0.16	
PET	Same imaging protocol	144	167	None			

When analyzing the CT data, inclusion of data from Aerts et al. [6] substantially affected the results. Although the MD Anderson data set was large, no radiomics feature was produced from the modeling process that was also significant in the independent testing data. However, inclusion of Aerts et al. [6] data produced two radiomics features that were also significant in the independent testing data and an AUC above 0.7, as discussed at the beginning of the results presented here.

Examining subgroups of only HPV positive, HPV negative/unknown, or oropharyngeal cancer patients did not improve these results. The information on the covariates selected, the hazard ratio, 95% confidence interval, p value, and the AUC for these patient cohorts can be found in the Supplemental Material.

### PET patients

**Fig 2:**
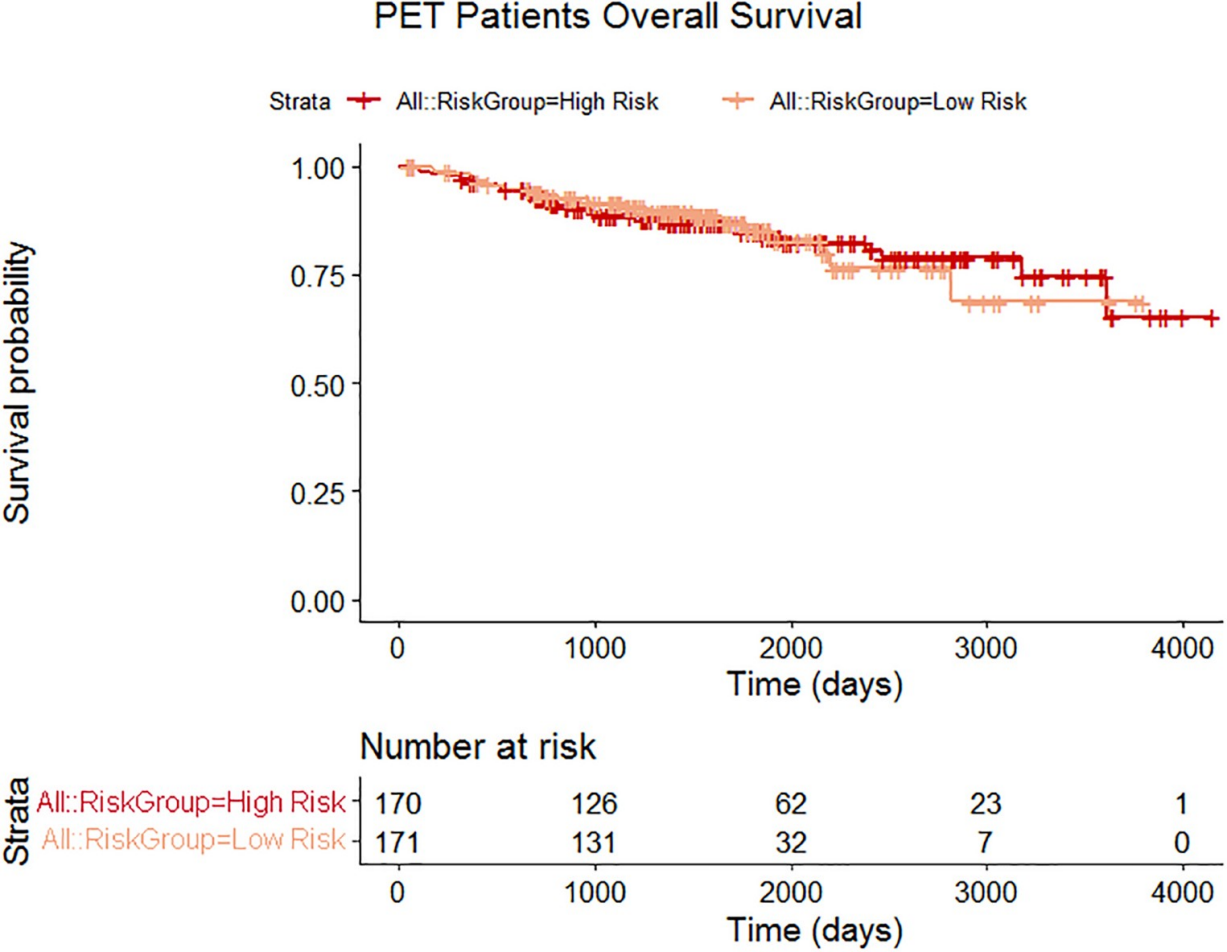
Patient survival curves using PET patient data. The independent testing data were from 341 patients who were assigned to High Risk or Low Risk groups according to prediction scores from the Cox model fit using the training data and two covariates: HPV status and coarseness calculated with use of using 64 gray levels. The High Risk and Low Risk groups were not statistically separated as shown by the overlap of the survival curves. These patient curves are called “All” and are in red and orange. For the subset of patients with the same imaging protocol, no covariates were selected, therefore, the patients could not be separated into High Risk and Low Risk and no curves are displayed for the subset patient group.

When using the whole patient cohort, HPV status was selected from the forward selection of the clinical variables. Four radiomics features had a p-value < 0.01 when HPV status was held within the Cox proportional hazards model. Three covariates were selected from the bootstrap Lasso. The final selected model contained two covariates: HPV status and coarseness calculated using 64 gray levels. The AUC of this model on the independent testing data was 0.59. However, neither of the covariates was significant (p = 0.69, p = 0.16) when the Cox model was fit using the independent testing data or when selecting only one covariate. The High Risk and Low Risk groups were not statistically separated, as shown by the survival plots for these patients in Fig 2 where the curves overlap.

The subsets and iterations in a PET imaging protocol are known to affect radiomics features measured from PET images [20–24]. Therefore, including only patients scanned on a GE scanner with 20 or 21 subsets and two iterations reduced the training data to 144 patients and the independent testing data to 168 patients. These patients were imaged on Discovery ST, Discovery STE, or Discovery RX PET scanners which are all non-time of flight and did not model point spread function. The final model included no covariates, even when relaxing the p-value for passing the additional prescreening univariate Cox analysis, so this attempt to control for imaging parameters was not effective. Table 2 summarizes the model information for the whole patient data set and the subset of patients with the same imaging protocol.

Examining subgroups of only HPV positive, HPV negative/unknown, or oropharyngeal cancer patients did not improve these results. The information on the covariates selected, the hazard ratio, 95% confidence interval, p value, and the AUC for these patient cohorts is in the Supplemental Material.

## Discussion

In this study, we investigated radiomics features for HNSCC patients by using CT and PET images. Both studies included more than 600 patients. A recent study published by Orlhac et al. found that since the first published PET radiomics study in 2009, almost 80% of studies have included fewer than 100 patients [21]. Similarly, CT head and neck cancer studies often include about 200 patients. Our study included more than three times this amount for both CT and PET analyses.

We hypothesized that outcome models built with data from patients on controlled imaging protocols should perform better than models built with data from a varied patient cohort since the noise from imaging variability is removed in the former model. We were unable to demonstrate an improvement in prediction accuracy in a subset of patients with the same imaging protocol compared to a patient cohort with different imaging protocols. Across both types of patient groups we found that while some features selected were significant in both the training and testing cohorts, none of our analyses in CT or PET studies could find a reliable radiomics feature that correlated with overall survival that was better than tumor volume. Our negative results are in contrast to other radiomics studies of head and neck cancer patients. Other studies have found radiomics features that correlated with overall survival, locoregional control, and freedom from distant metastases [6, 9, 10, 14, 36, 37]. We also investigated these other outcomes, in addition to overall survival, and found similar results to the overall survival results presented here. We chose to focus on overall survival since there were more events which typically results in better model building. However, most of our patients had oropharyngeal cancer, whereas most other radiomics studies included patients with general head and neck cancers that included sites such as the larynx. Foy et al. also showed that there are differences in implementation of the various radiomics software tools [38]. All of these differences could contribute to the differences in results found in other studies compared to our study.

We attempted many manipulations of the data, including the patient cohorts all one HPV status (e.g., positive), which removed the issue of HPV status having different rates of survival, and radiomics features were not consistently correlated with survival. Different splitting techniques of the training and testing cohorts yielded similar, negative, results. For example, all patients were assigned a number based on their row in the data table, then using this number (and not their MRN), patients were split based on an even or odd number. The data between the training and testing was similar in patient number, HPV status composition, event rate, and other patient demographics regardless of splitting technique. Based on this we are certain that the splitting technique used in the study did not affect the negative results found.

The complete CT patient cohort included the MAASTRO data, while the subset of patients with the same imaging protocol did not include any MAASTRO data. We were able to find a significant radiomics feature when the MAASTRO data was included, but when only MD Anderson data was included, no significant radiomics feature was found in the final model. As the subset of patients with the same imaging protocol only included MD Anderson patients, this could be a potential reason that an increase in prediction accuracy was not observed. The positive results from the inclusion of the MAASTRO data is consistent with the positive results found by Aerts et al. [6] in their original study with these data. Patient demographics (e.g., age, stage) were similar between the Aerts et al. [6] data set and the MD Anderson data set; however, there was a difference in event rate. This, in addition to the substantially different radiomics results between the two patient cohorts, indicated that there may have been differences in the patient population that we cannot understand through this study, such as differences in the patient population as it relates to the overall health care system. This suggests that radiomics signatures may not always be transferrable due to unseen differences in patient populations.

These discrepancies were not observed with the PET data. The MD Anderson and TCIA data sets were similar in patient demographics and event rate. These data sets produced no AUC above 0.6, even when sources of noise, such as imaging protocol or HPV status, were removed. Some of these models resulted in no covariates selected, which meant that even the two included clinical variables in the first stages of the modeling were not good predictors of survival. Since PET scans are not part of the standard of care for HNSCC patients, the patient cohort in this study that underwent PET scans may not be representative of HNSCC patients in general. This could explain why the traditional strong clinical correlates of survival, tumor volume and HPV status, were not selected or significant.

There are several limitations to this study. First, there are known clinical factors that affect survival that were not included in the analysis, such as smoking pack-years. The focus of this study was to demonstrate improvement in patient outcome models when imaging protocols are controlled, not to build the best possible outcome model that would include these clinical factors. Also, in common with other radiomics studies, only the primary GTV was analyzed. In patients with HNSCC, often nodes are involved, and some nodes may be irradiated due to suspected tumor involvement without definitive confirmation on images. It is difficult to determine how to best include these data in a conventional radiomics study such as this one. Deep learning approaches have shown promising results as a different technique to radiomics studies and may handle these challenges better [39, 40]. Additionally, most patients included in this study had oropharyngeal carcinoma. Therefore, applying the results from this study to other head and neck sites must be done with caution. This study was based on one software that has been successfully used in the past [1–3]. It is possible that other features not explored in this analysis may be more successful.

While the results of this particular study are negative, they highlight the areas that radiomics research should go towards in head and neck cancer patients. Our large CT study showed that the noise due to different imaging protocols can be overshadowed by noise due to differences between patient cohorts. This needs to be considered and investigated when applying radiomics signatures to patient groups from different regions with potentially different characteristics. Another avenue for CT radiomics for head and neck cancer may be analyzing the nodal regions as a recent paper has shown that analyzing the nodal regions can be used to predict distant metastases [41]. Additionally, for PET, the lack of any texture signature correlation with overall survival outweighed the noise due to different imaging protocols. This again identifies an avenue for future studies as alternative approaches are needed, for example, deep learning or development of PET-specific features. Lastly, we showed that harmonizing imaging protocols does reduce some uncertainties in radiomics features. Reducing this source of uncertainty should make it easier to investigate other sources of uncertainty (such as differences in patient cohorts) that impact the results of radiomics studies. If these additional sources of uncertainty can also be reduced, then this harmonization of imaging protocols could result in more precise radiomics studies.

## Conclusions

This is one of the largest radiomics studies in head and neck cancer patients and one of the largest PET radiomics studies in general. CT and PET-based radiomics features failed to improve survival models for head and neck cancer patients. Controlling the imaging protocol to minimize image uncertainties did not improve the radiomics models. The inconsistent CT findings here demonstrate that radiomics signatures for head and neck cancer patients may not be robust or reproducible, even when patient cohorts appear to be very similar. Head and neck cancer patient images may not have enough PET texture to be used in conventional radiomics studies.

## Supporting information

S1 Table Radiomics features used in CT analysis. (PDF)

S2 Table Radiomics features used in PET analysis. (PDF)

S3 Table Results of CT patient models. (PDF)

S4 Table Results of PET patient models. (PDF)

S1 Data (XLSX)
