# Supplementary material for: Radiomics features of the primary tumor fail to improve prediction of overall survival in large cohorts of CT- and PET-imaged head and neck cancer patients
Source: PLoS One. 2019 Sep 19;14(9):e0222509. doi: 10.1371/journal.pone.0222509 (PMC6752873; doi:10.1371/journal.pone.0222509)
Supplement: S2 Table — (PDF) [file pone.0222509.s002.pdf]

S2 Table. Radiomics Features used in PET Analysis

Gray Level Co-occurrence Matrix	Gray Level Run Length Matrix	Intensity Histogram	Neighborhood Gray Tone Difference Matrix
Auto Correlation	Gray Level Nonuniformity	Energy	Busyness
Cluster Prominence	High Gray Level Run Emphasis	Entropy	Coarseness
Cluster Shade	Long Run Emphasis	Kurtosis	Complexity
Cluster Tendency	Long Run High Gray Level Emphasis	Maximum	Contrast
Contrast	Long Run Low Gray Level Emphasis	Mean	Texture Strength
Correlation	Low Gray Level Run Emphasis	Median	
Difference Entropy	Run Length Nonuniformity	Minimum	
Dissimilarity	Run Percentage	Skewness	
Energy	Short Run Emphasis	Standard Deviation	
Entropy	Short Run High Gray Level Emphasis	Uniformity	
Homogeneity	Short Run Low Gray Level Emphasis	Variance	
Homogeneity 2			
Information Measure Correlation 1			
Information Measure Correlation 2			
Inverse Difference Moment Norm			
Inverse Difference Norm			
Inverse Variance			
Max Probability			
Sum Average			
Sum Entropy			
Sum Variance			
Variance			
