# Supplementary material for: Radiomics features of the primary tumor fail to improve prediction of overall survival in large cohorts of CT- and PET-imaged head and neck cancer patients
Source: PLoS One. 2019 Sep 19;14(9):e0222509. doi: 10.1371/journal.pone.0222509 (PMC6752873; doi:10.1371/journal.pone.0222509)
Supplement: S3 Table — (PDF) [file pone.0222509.s003.pdf]

S3 Table. Results of CT Patient Models

Patient Information			Model Information					Evaluation Information	
**Subset of Patients**	**Patients in training**	**Patients in testing**	**Covariates in final model**	**Hazard ratio of covariates on training data (95% CI)**	**Coefficient**	**Pearson correlation with volume**	**Pearson p-value**	**p-value of covariates when fit on testing data**	**AUC on testing data**
All patients	377	349	Volume	1.01 (1.00 – 1.02)	0.0096	N/A	N/A	p = 0.027	0.72
			HPV status	1.93 (1.27 – 2.95)	0.66	N/A	N/A	p = 0.018	
			Gray level nonuniformity (GLCM) calculated using thresholding and bit depth resampling	9.74×10^{-8} (9.22×10^{-12} – 1.03×10^{-3})	-16	-0.24	$p = 1.8 \times 10^{-6}$	p = 0.024	
			Inverse difference norm (GLCM) calculated using thresholding	3.34×10^{6} (13.5 – 8.28×10^{11})	15	0.46	$p < 2.2 \times 10^{-16}$	p = 0.017	
Same imaging protocol	260	251	HPV status	2.27 (1.32 – 3.89)	0.82	N/A	N/A	p = 0.79	0.55
			Cluster tendency (GLCM) calculated using thresholding,	1.07 (1.04 – 1.11)	0.072	0.22	p = 0.00035	p = 0.90	

			smoothing, and bit depth resampling						
Same imaging protocol HPV positive	168	152	Complexity (NGTDM) calculated using thresholding and smoothing	1 (1 – 1)	1.89 x 10^{-6}	0.35	p = 3.1 x 10^{-6}	p = 0.16	0.65
Same imaging protocol HPV negative	92	99	Volume	1.02 (1.00 – 1.03)	0.018	N/A	N/A	p = 0.001	0.62
HPV positive	224	189	Volume<br><br>Complexity (NGTDM) calculated using thresholding	1.04 (1.02 – 1.06)<br>1.00 (1.00 – 1.00)	0.034<br><br>7.8 x 10^{-7}	N/A<br><br>0.32	N/A<br><br>p = 8.1 x 10^{-7}	p = 2.1 x 10^{-4}<br><br>p = 0.26	0.75 (volume alone 0.76)
HPV negative	153	160	Volume<br><br>Sum entropy (GLCM) calculated using thresholding	1.01 (1.01 – 1.02)<br>2.63 (1.51 – 4.61)	0.012<br><br>0.97	N/A<br><br>0.20	N/A<br><br>p = 0.013	p = 3.6 x 10^{-4}<br><br>p = 0.021	0.65 (volume alone 0.72)
Oropharynx	362	324	Volume<br><br>HPV status<br><br>Contrast (GLCM)	1.01 (1.01 – 1.02)<br>2.11 (1.37 – 3.26)<br>1.00 (1.00 – 1.00)	0.010<br><br>0.75<br><br>7.7 x 10^{-4}	N/A<br><br>N/A<br><br>0.037	N/A<br><br>N/A<br><br>p = 0.48	p = 0.013<br><br>p = 0.19<br><br>p = 0.94	0.69 (volume alone 0.71)

			calculated using thresholding<br><br>Information measure correlation 2 (GLCM) calculated using thresholding, smoothing, and bit depth resampling	 <br><br><br>460 (4.1 – 5.2 x 10^4)	 <br><br><br>6.1	 <br><br><br>0.42	 <br><br><br>$p < 2.2 \times 10^{-16}$	 <br><br><br>p = 0.060	
Oropharynx HPV positive	224	189	Volume<br><br>Complexity (NGTDM) calculated using thresholding	1.04 (1.02 – 1.06)<br>1.00 (1.00 – 1.00)	0.034<br><br>7.8 x 10-7	N/A<br><br>0.32	N/A<br><br>$p = 8.1 \times 10^{-7}$	$p = 2.1 \times 10^{-4}$<br><br>p = 0.26	0.75 (volume alone 0.76)
Oropharynx HPV negative	138	135	Volume<br><br>Sum entropy (GLCM) calculated using thresholding	1.01 (1.01 – 1.02)<br>3.09 (1.65 – 5.79)	0.012<br><br>1.12	N/A<br><br>0.27	N/A<br><br>p = 0.0014	p = 0.012<br><br>p = 0.13	0.64 (volume alone 0.68)
