# Supplementary material for: Radiomics features of the primary tumor fail to improve prediction of overall survival in large cohorts of CT- and PET-imaged head and neck cancer patients
Source: PLoS One. 2019 Sep 19;14(9):e0222509. doi: 10.1371/journal.pone.0222509 (PMC6752873; doi:10.1371/journal.pone.0222509)
Supplement: S4 Table — (PDF) [file pone.0222509.s004.pdf]

S4 Table. Results of PET Patient Models

Patient Information			Model Information					Evaluation Information	
**Subset of Patients**	**Patients in training**	**Patients in testing**	**Covariates in final model**	**Hazard ratio of covariates on training data (95% CI)**	**Coefficient**	**Pearson correlation with volume**	**Pearson p-value**	**p-value of covariates when fit on testing data**	**AUC on testing data**
All patients	345	341	HPV status<br><br>Coarseness (NGTDM) calculated using 64 gray levels	1.8 (1.14 – 2.9)<br><br>2614 (11.6 – 5.9×10^5)	0.60<br><br>7.9	N/A<br><br>0.82	N/A<br><br>$p < 2.2 \times 10^{-16}$	p = 0.069<br><br>p = 0.16	0.59
Same imaging protocol	144	167	None						
Same imaging protocol HPV positive	117	137	None						
Same imaging protocol HPV negative	27	30	None						
HPV positive	207	206	Coarseness (NGTDM) calculated using 64 gray levels<br><br>Sum average (GLCM)	2.3×10^4 (6.6 – 8.3×10^7)<br><br>0.94 (0.89 – 0.99)	10.1<br><br>-0.064	0.82<br><br>0.0048	$p < 2.2 \times 10^{-16}$<br><br>p = 0.95	p = 0.28<br><br>p = 0.85	0.55

			calculated using 64 gray levels						
HPV negative	138	135	None						
Oropharynx	318	310	HPV status<br><br>Coarseness (NGTDM) calculated using fixed bin width	1.9 (1.2 – 3.1)<br><br>5.9 (2.5 – 13.8)	0.64<br><br>1.8	N/A<br><br>0.43	N/A<br><br>$p = 4.4 \times 10^{-16}$	$p = 0.61$<br><br>$p = 0.20$	0.58
Oropharynx HPV positive	206	206	Coarseness (NGTDM) calculated using 64 gray levels	3.38×10^5 (90.8 – 1.26×10^9)	13	0.82	$p < 2.2 \times 10^{-16}$	$p = 0.28$	0.59
Oropharynx HPV negative	112	104	None						
